# Effect of number of medications and complexity of regimens on medication adherence and blood pressure management in hospitalized patients with hypertension

**DOI:** 10.1371/journal.pone.0252944

**Published:** 2021-06-10

**Authors:** Eri Wakai, Kenji Ikemura, Chika Kato, Masahiro Okuda

**Affiliations:** 1 Department of Integrative Pharmacology, Mie University Graduate School of Medicine, Tsu, Mie, Japan; 2 Department of Pharmacy, Osaka University Hospital, Suita, Osaka, Japan; 3 Department of Pharmacy, Mie University Hospital, Tsu, Mie, Japan; Maastricht University Medical Center, NETHERLANDS

## Abstract

**Introduction:**

Good adherence of antihypertensives is recommended for the accomplishment of hypertension therapy. The number of medications and characteristics contributing to medication regimen complexity, such as dosage forms and dosing frequency, are known to influence medication adherence. However, the effect of medication regimen complexity on the therapeutic efficacy of medicines remains to be clarified. In the present study, we retrospectively investigated the effect of number of medications and medication regimen complexity on medication adherence and therapeutic efficacy in patients with hypertension.

**Methods:**

According to the inclusion and exclusion criteria, 1,057 patients, who were on medications including antihypertensives on admission at the Mie University Hospital between July 2018 and December 2018, were enrolled in this study. Poor blood pressure management was defined if the systolic or diastolic blood pressure were ≥140 mmHg or ≥ 90 mmHg. Medication regimen complexity was quantified using the medication regimen complexity index (MRCI) score.

**Results:**

Among 1,057 patients, 164 and 893 patients were categorized into poor and good adherence groups, respectively. The multivariate analyses revealed that age ≥ 71 years and oral MRCI score ≥ 19.5 but not number of oral medications were extracted as risk factors for poor medication adherence. Medication adherence and blood pressure management were poor in the group with oral MRCI score ≥ 19.5, regardless of the age. The rate of readmission was similar.

**Conclusion:**

Our study is the first to demonstrate that medication regimen complexity rather than number of medications is closely related to medication adherence and blood pressure management. Hence, physicians and/or pharmacists should consider the complexity of medication regimens while modifying them.

## Introduction

Hypertension is categorized as a non-communicable disease caused by unhealthy lifestyle choices, such as alcohol consumption [1] and physical inactivity [2], as well as diseases such as obesity [1, 2]. Hypertension increases the risk of developing cardiovascular disease [3]. The number of patients with hypertension was estimated to be 1.13 billion worldwide, as reported by the World Health Organization (WHO) in 2019 (https://www.who.int/health-topics/hypertension#tab=tab_1). Moreover, 7.5 million patients die from complications associated with hypertension (such as stroke and heart disease) every year [4]. Therefore, blood pressure management plays an important role in the prevention of cerebrovascular disease in patients with hypertension. Antihypertensive therapy, dietary modification, and exercise are standard strategies for management of hypertension [5, 6]. However, approximately 50% of patients receiving treatment for hypertension were unable to accomplish the target blood pressure goal as defined by guidelines for hypertension [7, 8].

Wetzels et al. [9] demonstrated that poor adherence to antihypertensive therapies worsened blood pressure management in patients with hypertension. Further, this systematic review indicated that the risk factors for poor medication adherence in patients with hypertension were age, cognitive impairment, cost of treatments, asymptomatic nature of hypertension, number of medications, and complexity of treatment. Thus, medication adherence is affected by both number of medications and characteristics contributing to the complexity of medication regimens, such as dosage forms and dosing frequency.

A standardized and validated tool (the medication regimen complexity index (MRCI)) has been developed to quantify the complexity of medication regimens [10]. High MRCI scores have been reported for elderly patients with heart failure and patients with chronic obstructive pulmonary disease [10, 11]. A systematic review reported that medication regimen complexity, quantified by MRCI scores, affected medication adherence and health outcomes, such as hospitalization and readmission [12]. In a study focusing on elderly patients with chronic kidney disease, patients on dialysis had high MRCI scores, but the relationship between medication adherence and MRCI score was not evaluated [13]. Further, there is little data on the effect of number of medications and the complexity of medication regimens on medication adherence and therapeutic efficacy of antihypertensives in patients with hypertension.

In the present study, we retrospectively investigated the relevance of number of medications and complexity of medication regimens for medication adherence and blood pressure management in patients with hypertension.

## Methods

### Patients and data collection

The data of scheduled inpatients (n = 1,126), who had medications including antihypertensives on admission at the Mie University Hospital between July 2018 and December 2018, were extracted from the electronic medical records. Patients were excluded if they had any missing data (n = 61) and if age < 20 years (n = 8). We collected data on patients’ characteristics (sex, age, body weight, body mass index (BMI), tobacco use, and alcohol use), biological parameters, and blood pressure on admission. To avoid overestimation of serum creatinine (Scr) levels due to the influence of patients’ muscle mass, value of Scr < 0.6 mg/dL was substituted for Scr = 0.6 mg/dL [14], and estimated glomerular filtration rate (eGFR) was calculated using eGFR (mL/min/1.73 m^2^) = 194 × age^−0.287^ × Scr^−1.094^ × 0.739 (if female) [15]. eGFR (mL/min) = eGFR (mL/min/1.73 m^2^) × body surface area/1.73 (m^2^).

### Evaluation of the MRCI scores for medications brought into the hospital by patients

The medication regimen complexity for those brought into the hospital on admission was quantified using the MRCI score [10]. This score consists of the following three sections: A (dosage forms), B (dosing frequency), and C (additional directions). The MRCI score was calculated as total score in each section. Number of oral medications and oral MRCI score were calculated solely for oral medications, excluding topical agents and self-injection device for insulin.

### Evaluation of medication adherence and blood pressure management

Pharmacists conducted the first interview for patients and assessed the patients’ medication adherence in brought medicines immediately on admission. Patients’ medication adherence was defined as poor if they could not manage medications by themselves or if they required their family caregivers and/or nurses to manage their medications. Further, poor blood pressure management was defined as having systolic or diastolic blood pressure ≥ 140 mmHg or ≥ 90 mmHg, respectively, on the day of admission, in accordance with the Japanese Society of Hypertension Guidelines for the Management of Hypertension (JSH 2019) [16].

### Evaluation of readmission patients

Patients who were rehospitalized at least once due to cardiovascular complications after hospital discharge, were defined as readmission patients.

### Statistical analyses

Statistical comparisons between two groups were performed using the Mann-Whitney U test and the Fisher’s exact test for continuous and categorical variables, respectively. Cut-off values of continuous variables were determined by receiver operating characteristics (ROC) curve method with JMP^®^ Pro version 14.3.0 (SAS Institute Inc, Cary, NC, USA). Multivariate analysis was performed to evaluate risk factors for poor medication adherence with following variables; age ≥ 71 years, sex, eGFR, number of oral medications ≥ 7, oral MRCI score ≥ 19.5, diabetes mellitus, heart failure, and alcohol use. Statistical analyses were performed with JMP^®^ Pro version 14.3.0. Statistical comparisons among four groups (age < 71 years and oral MRCI score < 19.5 group, age < 71 years and oral MRCI score ≥ 19.5 group, age ≥ 71 years and oral MRCI score < 19.5 group, and age ≥ 71 years and oral MRCI score ≥ 19.5 group) were analyzed by Fisher’s exact test with Bonferroni correction. Significance was established at a *p* value < 0.05 (Bonferroni correction; *p* value < 0.013).

### Ethics approval and consent to participate

This study was conducted in accordance with the Declaration of Helsinki and was approved by the Ethics Committee of Mie University Graduate School of Medicine and Faculty of Medicine (No. H2019-136). Informed consent was obtained through an opt-out method from all participants because the data were collected retrospectively from electronic medical records.

## Results

### Comparison of patient characteristics between the good and the poor adherence groups

According to the inclusion and exclusion criteria, 1,057 of 1,126 patients were enrolled in the present study. Table 1 shows the comparison of patient characteristics between the good and the poor adherence groups. The systolic and diastolic blood pressure, age, and blood urea nitrogen (BUN) were significantly higher in the poor adherence group than in the good adherence group. Further, the eGFR and rate of alcohol use were significantly lower in the poor adherence group than in the good adherence group. There were no significant differences in other characteristics between the good and the poor adherence groups.

**Table 1:** Comparison of patient characteristics between the good and the poor adherence groups.

Patient characteristics	Good adherence (n = 893)	Poor adherence (n = 164)	*p* value
Female	356 (40)	60 (37)	0.438
Age (years)	70 [21–94]	74 [22–89]	<0.001
Body weight (kg)	61 [32.3–133]	59 [25.7–146]	0.124
BMI	23.5 [13.8–47.4]	23.1 [16.1–58.1]	0.529
Tobacco use	406 (45)	84 (51)	0.175
Alcohol use	376 (42)	53 (32)	0.020
Diabetes mellitus	166 (19)	37 (23)	0.069
Heart failure	36 (4)	12 (7)	0.053
Baseline biological parameters			
AST (U/L)	26 [7–1247]	26 [8–231]	0.595
ALT (U/L)	22 [3–563]	21 [5–335]	0.300
γ-GTP (U/L)	32 [2–1127]	32 [2–927]	0.764
T-Bil (mg/dL)	0.8 [0.2–29]	0.7 [0.3–2.7]	0.066
BUN (mg/dL)	17.8 [5.2–97.7]	21.6 [5.0–93.9]	<0.001
eGFR (mL/min)	63 [3–124]	51 [3–128]	<0.001
Systolic blood pressure (mmHg)	131 [50–197]	141 [82–197]	<0.001
Diastolic blood pressure (mmHg)	73 [35–124]	75 [45–115]	0.035

Values are presented as median [range] or number (%).

Fisher’s exact test or Mann-Whitney U test was performed. ALT: alanine transaminase; AST: aspartate transaminase; BMI: body mass index; BUN: blood urea nitrogen; eGFR: estimated glomerular filtration rate; γ-GTP: gamma-glutamyl transpeptidase; T-Bil: total bilirubin

### Comparisons of number of medications and MRCI score between the good and the poor adherence groups

Table 2 shows the comparisons of number of medications and MRCI score between the good and the poor adherence groups. The median number of total medications, antihypertensives, and oral medications in the poor adherence group were significantly higher than those in the good adherence group (*p* < 0.001). Similar results were observed for the MRCI score of total and oral medications (*p* < 0.001). Next, we investigated the percentage contribution of each section to oral MRCI score. We found that the contribution of section B to oral MRCI score was higher than that of the other sections; the average contribution of sections A, B, and C were 9%, 51%, and 40%, respectively. A list of highest dosing frequencies in section B and examples for low MRCI and high MRCI score are shown in S1 and S2 Tables in S1 File, respectively.

**Table 2:** Comparisons of number of medications and MRCI score between the good and the poor adherence groups.

	Good adherence (n = 893)	Poor adherence (n = 164)	*p* value
Number of medications			
Total medications	7 [1–22]	9 [1–24]	<0.001
Oral medications	6 [1–22]	8 [1–22]	<0.001
Antihypertensives	1 [1–5]	2 [1–4]	<0.001
MRCI score			
Total medications	16 [3–75.5]	25 [3–63]	<0.001
Oral medications	15 [3–66]	23 [3–63]	<0.001

Values are presented as median [range].

Mann-Whitney U test was performed.

### Evaluation of risk factors for the poor medication adherence by multivariate analysis

Multivariate analysis was conducted to investigate the risk factors for the poor medication adherence (Table 3). As shown in S1 Fig in S1 File, the cut-off values (area under the ROC curve: AUC) of the age, number of oral medications, and oral MRCI score for poor adherence were 71 (0.62), 7 (0.70), and 19.5 (0.71), respectively. Multivariate analysis revealed that the independent risk factors for poor medication adherence were age ≥ 71 years (odds ratio (OR): 2.025, *p* < 0.001) and oral MRCI score ≥ 19.5 (OR: 2.911, *p* < 0.001). On the other hand, number of oral medications was not a significant risk factor for medication adherence.

**Table 3:** Multivariate logistic regression analyses for poor medication adherence in patients who had medications including antihypertensives on admission.

Variables	Odds ratio	95% CI	*p* value
Female	1.475	0.989–2.199	0.057
Age ≥ 71 years	2.025	1.397–2.936	<0.001
eGFR (mL/min)	0.998	0.992–1.004	0.526
Number of oral medications ≥ 7	1.544	0.895–2.664	0.119
Oral MRCI score ≥ 19.5	2.911	1.730–4.898	<0.001
Diabetes mellitus	1.416	0.939–2.135	0.097
Heart failure	1.601	0.782–3.276	0.198
Alcohol use	1.425	0.948–2.142	0.088

CI: confidence interval, eGFR: estimated glomerular filtration rate.

### Relevance of age and complexity of medication regimens for medication adherence and blood pressure management

**Fig 1:**
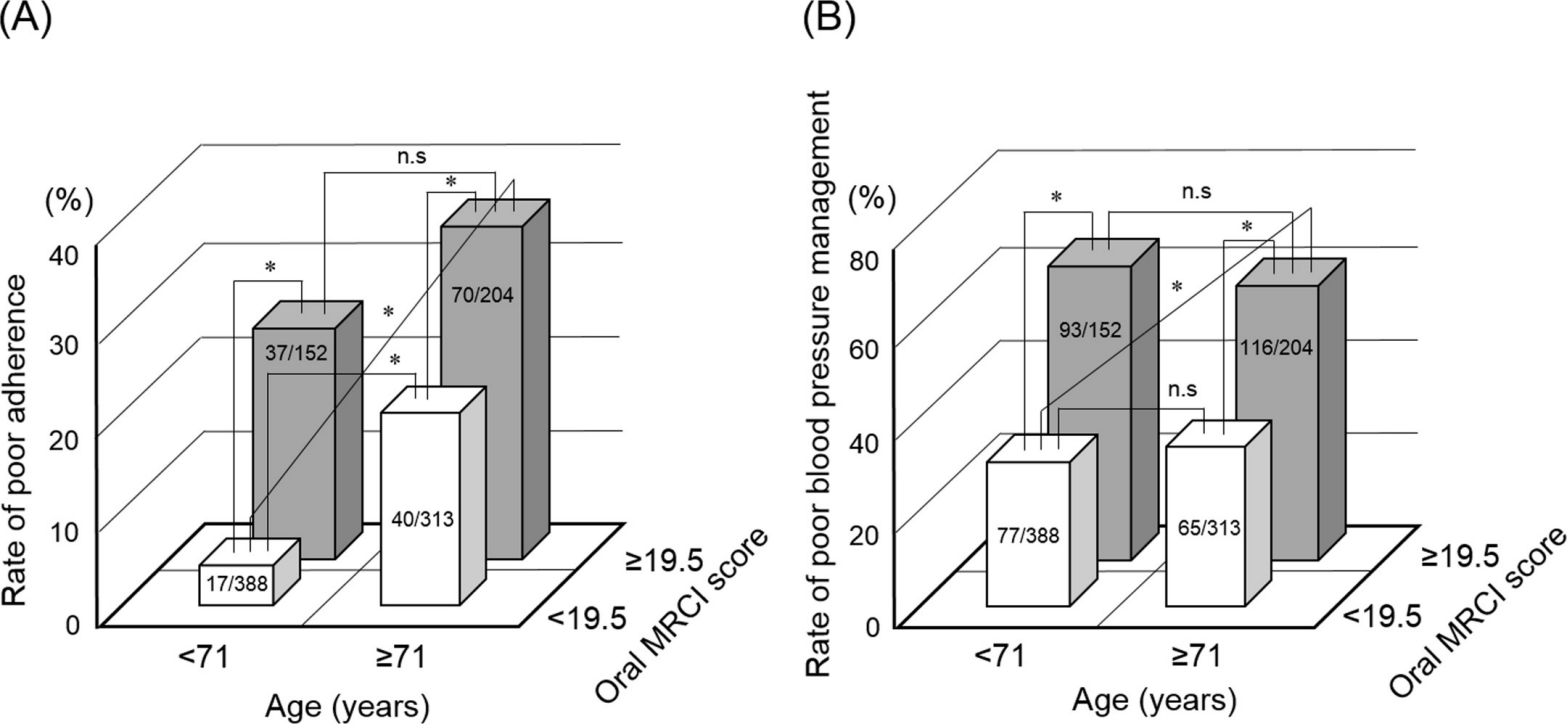
Relevance of age and oral MRCI score for medication adherence and blood pressure management. Comparisons of poor adherence rate (A) and poor blood pressure management rate (B) were performed among four groups. Statistical analyses were performed using Fisher’s exact test with Bonferroni correction. Values in the columns are presented as patients’ number. *p < 0.013, n.s: not significant.

We categorized patients into four groups based on the cut-off values of the age and oral MRCI score. Fig 1 shows the comparisons of poor adherence rate (A) and poor blood pressure management rate (B) among four groups. As shown in Fig 1A, in the group with oral MRCI score < 19.5, the rate of poor adherence with age ≥ 71 years was significantly higher than that with age < 71 years (13% vs 4%, *p* < 0.001). However, there was no significant difference between age < 71 years and age ≥ 71 years in the group with oral MRCI score ≥ 19.5 (24% vs 34%, *p* = 0.043). The rate of poor adherence in the group with oral MRCI score ≥ 19.5 was significantly higher than that with oral MRCI score < 19.5, regardless of the age (24% vs 4% with age < 71 years, *p* < 0.001, 34% vs 13% with age ≥ 71 years, *p* < 0.001). Similar results were observed for the rate of poor blood pressure management, as shown in Fig 1B. However, there was no significant difference between the groups with age < 71 years and age ≥ 71 years in the groups with oral MRCI score < 19.5 (20% vs 21%, *p* = 0.759).

### Relevance of age and oral MRCI score for readmission rate

**Fig 2:**
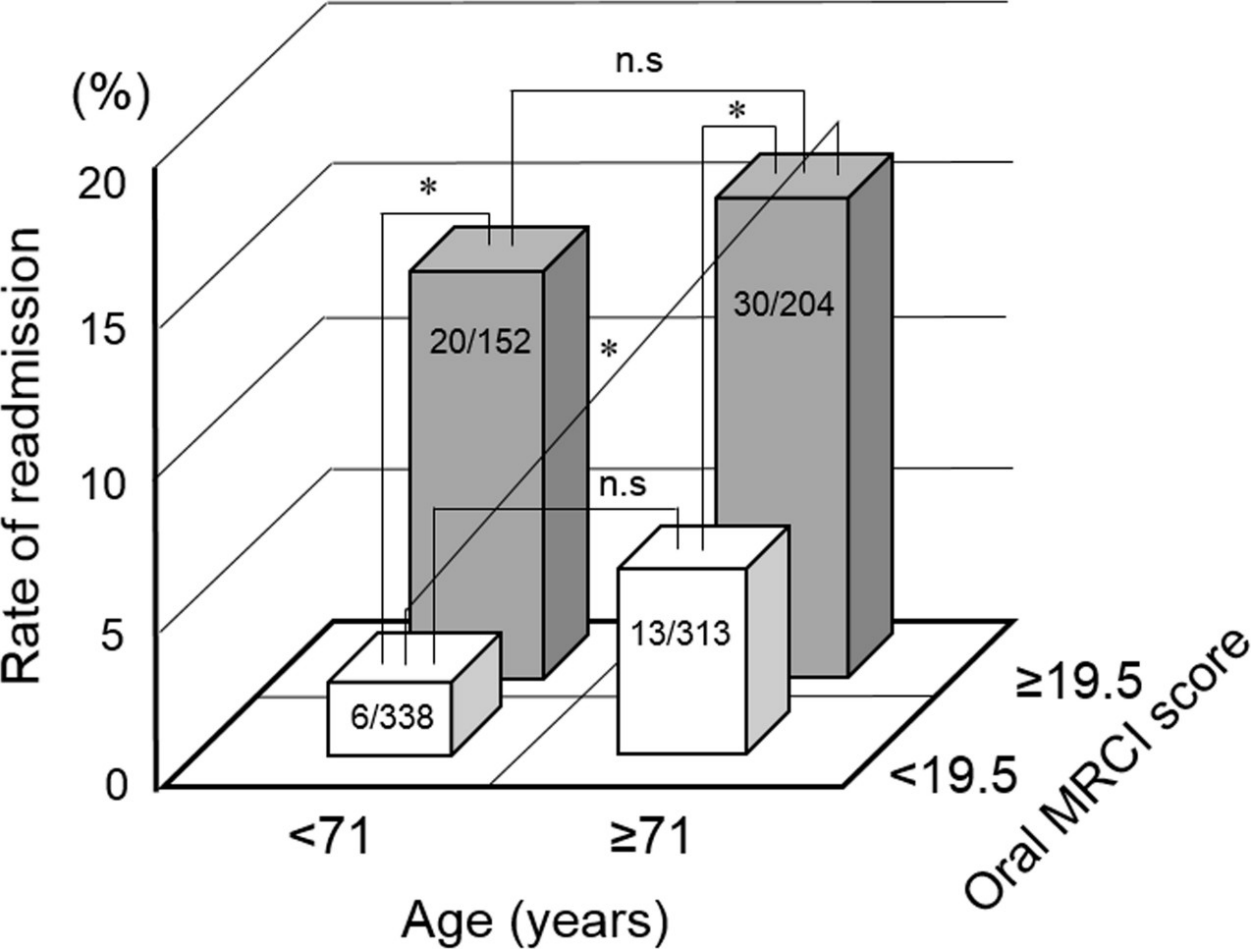
Relevance of age and oral MRCI score for readmission rate. The comparisons of readmission rate were performed among four groups. Statistical analyses were performed using Fisher’s exact test with Bonferroni correction. Values in the columns are presented as patients’ number. *p < 0.013, n.s: not significant.

Furthermore, we investigated the relevance of age and oral MRCI score for readmission rate. Fig 2 shows the comparisons of readmission rate between four groups. There was no significant difference between the groups with age < 71 years and age ≥ 71 years (2% vs 4% with oral MRCI score < 19.5, *p* = 0.086, 13% vs 15% with oral MRCI score ≥ 19.5, *p* = 0.692). However, the rate of readmission in the group with oral MRCI score ≥ 19.5 was significantly higher than in the group with oral MRCI score < 19.5, regardless of the age (2% vs 13% with age < 71 years, *p* < 0.001, 4% vs 15% with age ≥ 71 years, *p* < 0.001).

## Discussion

Currently, little is known about the effect of number of medications and complexity of medication regimens on medication adherence and blood pressure management. To our knowledge, this is the first study to report the relevance of number of medications and complexity of medication regimens for medication adherence and therapeutic efficacy in patients with hypertension.

Polypharmacy, defined as inappropriate prescriptions that are more than necessary, is known to be a global issue and has been one of the important causes of decreased medication adherence [17]. Moreover, polypharmacy increases the risk of adverse events and potential drug-drug interactions [18, 19]. There is limited information regarding the criteria for polypharmacy; however, prescription of ≥ 5–7 medications is defined as polypharmacy [18, 20]. In the present study, the cut-off value of number of medications for poor adherence was 7, which was similar to previous studies [18, 20]. Furthermore, the cut-off value of oral MRCI score for poor adherence was 19.5; this is the first report to indicate a criterion to predict poor adherence using the MRCI score in patients with hypertension.

A previous study reported the correlation between number of medications and MRCI score [21], similar results were observed in our study (S2 Fig in S1 File). Therefore, we further investigated differences in the correlation between number of medications and medication regimen complexity with medication adherence and blood pressure management. As shown in Table 3, multivariate analysis revealed that the independent risk factors for the poor medication adherence were age ≥ 71 years and oral MRCI score ≥ 19.5 but not number of oral medications. In addition, the rates of medication adherence and blood pressure management were poor in patients with high MRCI score (Fig 1). Therefore, these findings suggest that the medication regimen complexity rather than number of oral medications is related to medication adherence and blood pressure.

Our present study showed that patient age is also a risk factor for the poor medication adherence (Table 3). In general, medication adherence in elderly patients tends to be poor because of decreased physical functions and cognitive decline [22]. Moreover, renal function in the poor adherence group were lower than those in the good adherence group (Table 1). A previous study demonstrated that polypharmacy is a serious problem in elderly patients and increases the risk of adverse events such as acute kidney injury [22]. Several drugs are excreted from the kidney. Therefore, administration of unnecessary and excessive medications could cause renal failure in elderly patients [23].

In Japan, 33% of emergency hospitalizations are linked to drug overdose due to medication errors associated with multiple medications [24]. It is known that rate of readmission is high in elderly patients [25], and Abou-Karam N. et al [26] reported that the complexity of medication regimens increased the risk of readmission in patients with heart failure, acute myocardial infraction, pneumonia, or chronic obstructive pulmonary disease. However, as shown in Fig 2, the rates of readmission in the groups with ≥ 19.5 oral MRCI score were high, regardless of age < 71 years (Fig 2). Thus, these results demonstrate that complexity in medication regimens could increase the risk of readmission after discharge in patients with hypertension.

Our study has several limitations. First, this study was a retrospective study that included patients from a single institution and potential selection bias could not be excluded. Second, there is a possibility that patients’ adherence was not correctly evaluated, because adherence was assessed based on pharmacists’ judgment and not by quantification using adherence tools. However, we assumed that the evaluation of patients’ adherence to the medications was accurate because post admission evaluations of adherence by nurses were consistent with those by pharmacists. Third, we could not assess the unnecessary medications, which were not required for treatment, among the medications brought into the hospital by patients on admission. Thereby, the MRCI score and number of medications as reported in this study may be higher than their actual values. Fourth, it was difficult to examine the potential confounders such as presence of cognitive impairment and social factors because of the retrospective nature of this study. Because we focused on patients in an acute hospital of single-center, the external validity of this study may not be applicable in a chronic hospital. Future studies with a larger scale and multicenter prospective study should be conducted to evaluate the effect of age, number of medications, and medication regimen complexity on adherence and blood pressure management in patients with hypertension.

## Conclusions

Our study is the first to demonstrate that medication regimen complexity rather than number of medications is closely related to medication adherence and blood pressure. Hence, physicians and/or pharmacists should consider the complexity of medication regimens while modifying them. The present findings provide useful information for the achievement of therapeutic outcomes in patients with hypertension.

## Supporting information

S1 File (PDF)
