# Supplementary material for: Effect of number of medications and complexity of regimens on medication adherence and blood pressure management in hospitalized patients with hypertension
Source: PLoS One. 2021 Jun 10;16(6):e0252944. doi: 10.1371/journal.pone.0252944 (PMC8191947; doi:10.1371/journal.pone.0252944)
Supplement: S1 File — (PDF) [file pone.0252944.s001.pdf]

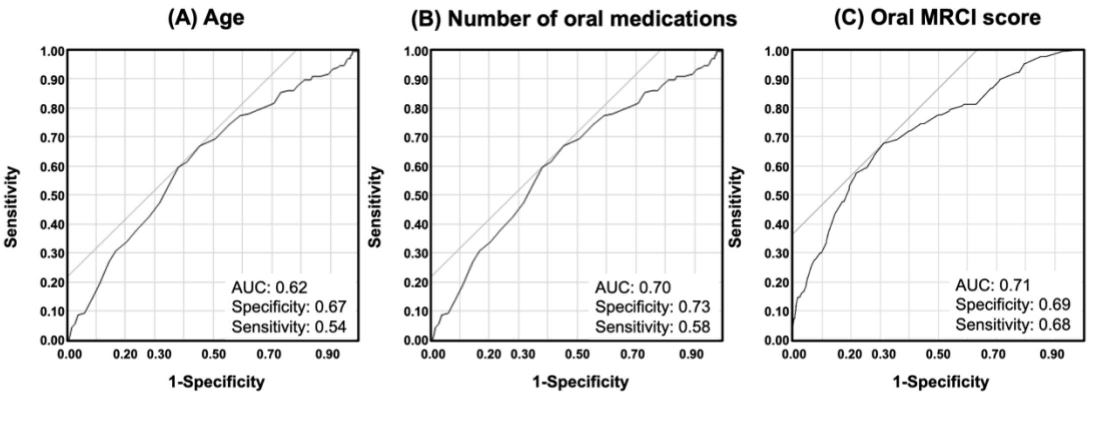

S1 Fig. The ROC curve of the age (A), number of oral medications (B), and oral MRCI score (C) for poor adherence.

Cut-off values of continuous variables were determined by receiver operating characteristics (ROC) curve method.

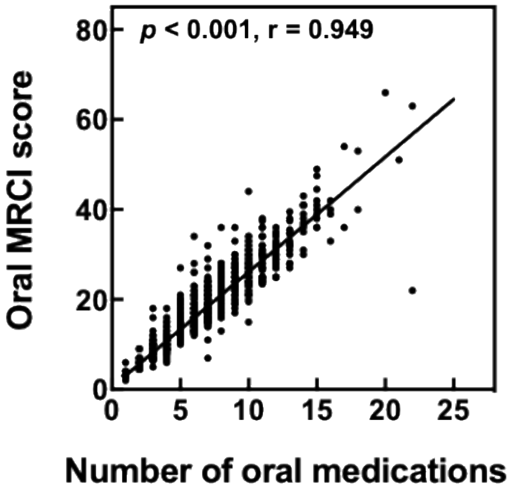

S2 Fig. Correlation between number of oral medications and oral MRCI score

Statistical analysis was performed using Spearman correlation coefficient. Each point represents a patient.

S1 Table. A list of highest dosing frequencies in section B

	Medicine
1	Acetaminophen
2	Adenine
3	Sodium Alginate
4	Alprazolam
5	Ambroxol
6	Benfotiamine, Pyridoxine Hydrochloride, Cyanocobalamin
7	Benzbromarone
8	Bifidobacterium Lactomin
9	L-Carbocisteine
10	Camostat Mesilate
11	Cefcapene Pivoxil Hydrochloride Hydrate
12	Dextromethorphan Hydrochloride Hydrate
13	Domperidone
14	Duloxetine Hydrochloride
15	Ethyl Icosapentate
16	Etizolam
17	Fluconazole
18	Ifenprodil Tartrate
19	Isosorbide
20	Lanthanum Carbonate Hydrate
21	Limaprost Alfadex
21	Loxoprofen Sodium Hydrate
23	Magnesium oxide
24	Mecobalamin
25	Mosapride Citrate Hydrate
26	Naproxen
27	Nicergoline
28	Pancrelipase
29	Phenobarbital
30	Phenytoin
31	Potassium Gluconate
32	Prochlorperazine Maleate
33	Rebamipide
34	Riociguat
35	Sildenafil Citrate
36	Sofalcone
37	Teprenone
38	Tocopherol Nicotinate
39	Tramadol Hydrochloride
40	Ursodeoxycholic acid
41	Clostridium butyricum
42	Voglibose

S2 Table. Examples of medication regimens in a patient with high MRCI score (A) and low MRCI score (B).

(A)

	Medicine	Dosing frequency	Oral MRCI score			
			A	B	C	Total
1	Lanthanum Carbonate Hydrate tablet	1 tablet, 3 times daily, right after meals	5	38	23	66
2	Bepotastine Besilate tablet	1 tablet, once daily, after meals				
3	Sofalcone powder	1 package, 3 times daily, after meals				
4	Furosemide tablet	1 tablet, once daily, after meals				
5	Teprenone capsule	1 capsule, 3 times daily, after meals				
6	Lomerizine Hydrochloride tablet	1 tablet, twice daily, after meals				
7	Vildagliptin tablet	1 tablet, once daily, after meals				
8	Nifedipine CR tablet	1 tablet, once daily, after meals				
9	Carvedilol tablet	1 tablet, once daily, after meals				
10	Alfacalcidol capsule	1 tablet, once daily, after meals				
11	Spironolactone tablet	1 tablet, once daily, after meals				
12	Pitavastatin Calcium Hydrate tablet	1 tablet, once daily, after meals				
13	Nalfurafine Hydrochloride tablet	1 tablet, once daily, after meals				
14	Loxoprofen Sodium Hydrate tablet	1 tablet, 3 times daily, after meals				
15	Rebamipide tablet	1 tablet, 3 times daily, after meals				
16	Sennoside tablet	2 tablets, once daily, at bedtime				
17	Magnesium Oxide tablet	1 tablet, 3 times daily, after meals				
18	Domperidone tablet	1 tablet, twice daily, before meals				
19	Lansoprazole tablet	1 tablet, once daily, after meals				
20	Sodium Alginate syrup	20 mL, 3 times daily, before meals				
21	Febuxostat tablet	1 tablet, once daily, after meals				
22	Aspirin tablet	1 tablet, once daily, after meals				

(B)

	Medicine	Dosing frequency	Oral MRCI score			
			A	B	C	Total
1	Amlodipine Besilate tablet	1 tablet, once daily, after meals	1	1	1	3

S3 Table. The values used to build graphs in Figure

Fig 1A groups	Rate of poor adherence (%)	Number of poor adherence patients	Number of all patients
Age < 71 years and MRCI < 19.5	4	17	388
Age < 71 years and MRCI ≥ 19.5	24	37	152
Age ≥ 71 years and MRCI < 19.5	13	40	313
Age ≥ 71 years and MRCI ≥ 19.5	34	70	204

Fig 1B groups	Rate of poor blood pressure management (%)	Number of poor blood pressure management patients	Number of all patients
Age < 71 years and MRCI < 19.5	20	77	388
Age < 71 years and MRCI ≥ 19.5	61	93	152
Age ≥ 71 years and MRCI < 19.5	21	65	313
Age ≥ 71 years and MRCI ≥ 19.5	57	116	204

Fig 2 groups	Rate of readmission (%)	Number of readmission patients	Number of all patients
Age < 71 years and MRCI < 19.5	2	6	388
Age < 71 years and MRCI ≥ 19.5	13	20	152
Age ≥ 71 years and MRCI < 19.5	4	13	313
Age ≥ 71 years and MRCI ≥ 19.5	15	30	204
